# *Bacillus* H47 triggers *Olea europaea* metabolism activating DOXP and shikimate pathways simultaneously and modifying leaf extracts’ antihypertensive activity

**DOI:** 10.3389/fmicb.2022.1005865

**Published:** 2022-10-04

**Authors:** Estrella Galicia-Campos, Ana García-Villaraco, M. B. Montero-Palmero, F. Javier Gutiérrez-Mañero, Beatriz Ramos-Solano

**Affiliations:** Facultad de Farmacia, Boadilla del Monte, Universidad San Pablo-CEU, CEU Universities, Madrid, Spain

**Keywords:** PGPB, secondary metabolism, oleuropein, flavonols, *Olea europaea*, plant adaptation, abiotic stress

## Abstract

Improvement of plant adaptation by beneficial bacteria (PGPB) may be achieved by triggering multiple pathways to overcome the environmental stress on plant’s growth cycle, activating plant’s metabolism. The present work reports the differential ability of three *Bacillus* strains to trigger olive tree metabolism, among which, only H47 was outstanding increasing iridoid and flavonol concentration. One-year old olive seedlings grown open air, under harsh conditions of water shortage in saline soils, were root-inoculated with three *Bacillus* PGPB strains throughout a 12-month period after which, photosynthesis was determined; photosynthetic pigments and bioactive secondary metabolites (iridoids and flavonols) were analyzed, and a study of gene expression of both pathways involved was undertaken to unravel molecular targets involved in the activation. All three strains increased plant fitness based on photosynthetic values, increasing energy dissipation capacity to lower oxidative stress; only H47 increased CO_2_ fixation and transpiration. *Bacillus* H47 was found to trigger synthases in the DOXP pathway (up to 5-fold in DOXP-synthase, 3.5-fold in Iridoid synthase, and 2-fold in secologanin synthase) associated to a concomitant increase in iridoids (up to 5-fold in oleuropein and 2-fold in its precursor secologanin). However, despite the 2-fold increases detected in the two predominant flavonols, gene expression was not enhanced, suggesting involvement of a pulse activation model proposed for innate immunity. Furthermore, the activity of leaf extracts to inhibit Angiotensin Converting Enzyme was evaluated, to explore further uses of plant debris with higher added value. Despite the increases in iridoids, leaf extracts from H47 did not increase ACE inhibition, and still, increased antihypertensive potential in oil obtained with this strain is to be explored, as leaves are the source for these metabolites which further translocate to fruits. In summary, *Bacillus* H47 is an effective strain to increase plant adaptation to dry and saline environments, activates photosynthesis and secondary metabolism in olive tree.

## Introduction

While growing within their natural habitat, plants are subjected to many different changes in their physical and biological surroundings. As sessile organisms, plants have developed an innate immune system and an active secondary metabolism to improve their adaptation to biotic and abiotic stress conditions (Oh et al., 2009). The plant immune system is activated in waves to cope with simultaneous changes in environmental parameters, usually combined with additional more persistent stress conditions, key for the overall fitness of the plant and its ability to survive rapid changes within its environment (Petridis et al., 2012; Mousavi et al., 2019); this has been termed as the pulse network response (Kollist et al., 2019). This adaptative metabolism demands energy and a strong carbon supply to build up carbon scaffoldings that are provided by photosynthesis, which is the key process for plant growth and survival. Furthermore, the fate of photosynthates has to be balanced between plant growth and adaptation so a good coordination for the best use of energetic resources is key for success (Mauch-Mani et al., 2017).

However, photosynthesis arrest may happen due to many factors including high light intensity, temperature, and water resource availability (Skodra et al., 2021). On one hand, the usual oxidative stress of light reactions is further enhanced under stress and needs to be counteracted by an active ROS scavenging system, allowing ROS within healthy levels for systemic metabolic coordination (Baxter et al., 2014). On the other hand, with low water availability, stomatal closure occurs as an early response to abscisic acid (ABA) produced by dehydrated roots (Grace, 2005), decreasing CO_2_ entrance and compromising C assimilation. As water stress is a relevant problem, plants have many innate mechanisms that regulate adaptation to stress.

In addition to plant’s genetic endowment to keep photosynthesis running, action has been taken to prevent the yield losses due to drought stress, and advances in agronomical practices, traditional breeding and modern biotechnological tools have been developed (Gill and Tuteja, 2010), aiming to improve adaptation of crops to drought stress (Sofo et al., 2004). Among these, beneficial bacteria have proved to be effective (Ennajeh et al., 2009; Oh et al., 2009), and appear as one of the most promising tools to achieve this goal, as they trigger multiple targets simultaneously (Ilangumaran and Smith, 2017) and allow fine-tunning of resources allocation (Mauch-Mani et al., 2017).

The term plant growth-promoting rhizobacteria was coined by Kloepper et al. (1980) to refer to free-living beneficial bacteria that inhabit the rhizosphere enhancing plant growth. Although the term has evolved to the more inclusive Plant Growth Promoting Bacteria (PGPB) to integrate beneficial bacteria from other origins, the mechanisms by which they improve plant fitness remain the same (Sayyed et al., 2016; Rosier et al., 2018). PGPB are able to improve plant nutrition, to biocontrol soil microorganisms, to modify plant metabolism altering hormonal balance, photosynthesis or secondary metabolism by systemic induction, as well as triggering plant immune system. Thus, the role of beneficial rhizobacteria to improve photosynthetic performance and trigger secondary metabolism simultaneously appears as a good alternative to increase the levels of bioactive secondary metabolites (Algar et al., 2013; Lucas et al., 2014; García-Cristobal et al., 2015; Garcia-Seco et al., 2015), protect against biotic and abiotic stress (Barriuso et al., 2008), and other frequent situations in agriculture (Gutiérrez-Mañero et al., 2015). Considering the multitarget capacity of PGPB strains (Ilangumaran and Smith, 2017) and the pulse network model response of plants immune system (Kollist et al., 2019), a wave-like activation triggered by bacteria on different targets appears as a likely model to happen.

*Olea europaea* is one of the most extended crops in the Mediterranean area, naturally endowed with mechanisms for high water use efficiency. The most characteristic secondary metabolites present in olive trees are iridoids, triterpenes, and phenolic compounds (flavonols), all conferring a high antioxidant potential (Khaliq et al., 2015; Skodra et al., 2021). Biosynthesis of these metabolites involves the DOXP-pathway for iridoids, the mevalonic acid pathway for triterpenes, and the shikimate-flavonol pathway for flavonols, which accumulate in fruits and also in leaves. Reported benefits of olive leaves include antihypertensive potential due to the coordinated effects of iridoids (oleuropein, oleacein, and ligustroside) and triterpenes (oleanolic acid; El Riachy et al., 2011; Alagna et al., 2012; Bulotta et al., 2013); antitumor potential has also been reported (Celano et al., 2019).

As the use of beneficial rhizobacteria capable of modulating secondary metabolism pathways of plants and improve adaptation to abiotic stress has proved efficient in different species, including olive tree (Algar et al., 2013; Lucas et al., 2014; García-Cristobal et al., 2015; Garcia-Seco et al., 2015; Galicia-Campos et al., 2020; Gutierrez-Albanchez et al., 2020), the present study reports effects of three *Bacillus* strains to obtain polyphenol enriched extracts with enhanced antihypertensive potential, to improve value of byproducts in line with the green pact of the EU. To achieve this objective, olive plantlets grown in high saline conditions were inoculated along 12 months, after which photosynthesis, photosynthetic pigments, total phenols, bioactive secondary metabolites (flavonols, the iridoids secologanoside, and oleouropein) were analyzed; finally, expression of key genes involved in bioactive synthesis was studied by RT-qPCR in the most effective strain, and *in vitro* antihypertensive activity was assessed.

## Materials and methods

### Beneficial strains and olive tree variety

The three beneficial strains (G7, L44, and H47) assayed in this study were Gram-positive sporulated bacilli isolated from the rhizosphere of *Pinus pinea* (L44) and *P. pinaster* (G7 and H47; Barriuso et al., 2005). They were able to produce siderophores (G7, H47) and auxins (L44). They have been identified by 16s rDNA sequencing as *Bacillus simplex* G7 (OP324816), *B. aryabhatai* L44 (OP324815), and *B. velezensis* H47 (OP324817).

*Olea europaea* (L) var. Arbequina plantlets were used for the study. Plantlets were bought from a local producer Lucena de Encinarejo S.L.(Córdoba).

### Inocula preparation and delivery to plants

Bacterial strains were maintained at −80°C in nutrient broth with 20% glycerol. Inocula were prepared by streaking strains from −80°C onto plate count agar (PCA) plates, incubating plates at 28°C for 24 h. Then, they were grown in Luria Broth liquid media (LB) under shaking (1,000 rpm) at 28°C for 24 h; inocula density was adjusted to 1 × 10^8^ cfu/ml and 500 ml were delivered to roots of each plant every 15 days from October 2017 to October 2018.

### Experimental design

Six-month olive plantlets were transplanted into 5 l pots with soil from the Guadalquivir Marshes. Plants were arranged in lines on an experimental plot within the marshes (37°06′34.5′′ N, 6°20′22.7′′ W); pot position was changed every 2 weeks to avoid side-effects. Plants were watered every 15 days. The electric conductivity of water and soil was 8.20 and of 6.07 dS/m, respectively.

Bacteria were root-inoculated by soil drench every 15 days from October 2017 to October 2018, so plants received 500 ml of water every week, alternating inoculum and water. Six plants per treatment were inoculated, being one bacterial strain a treatment, with three replicates (two plants each). Samples were taken in October 2018 and photosynthesis was measured (fluorescence and CO_2_ fixation). Leaves from two plants in each treatment were pooled before powdering in liquid nitrogen and constituted a replicate; powder was stored at −80°C till analysis. Photosynthetic pigments were determined as well as total phenols, flavonols, and iridoids as metabolic markers of the induction. Expression of genes involved in the biosynthesis of flavonols and iridoids were analyzed by RT-qPCR in the most effective strain and controls; finally, the potential antihypertensive effect of leaf extracts was evaluated *in vitro*, calculating its ability to inhibit Angiotensin Converting Enzyme as indicated in 3.10.

### Photosynthesis (chlorophyll fluorescence)

Photosynthetic efficiency was determined through the chlorophyll fluorescence emitted by photosystem II. Chlorophyll fluorescence was measured with a pulse amplitude modulated (PAM) fluorometer (Hansatech FM2, Hansatech, Inc., United Kingdom). After dark-adaptation of leaves, the minimal fluorescence (Fo; dark-adapted minimum fluorescence) was measured with a weak modulated irradiation (1 μmol m^−2^ s^−1^). Maximum fluorescence (Fm) was determined for the dark-adapted state by applying a 700 ms saturating flash (9,000 μmol m^−2^ s^−1^). The variable fluorescence (Fv) was calculated as the difference between the maximum fluorescence (Fm) and the minimum fluorescence (Fo). The maximum photosynthetic efficiency of photosystem II (maximal PSII quantum yield) was calculated as Fv/Fm. Immediately, the leaf was continuously irradiated with red-blue actinic beams (80 μmol m^−2^ s^−1^) and equilibrated for 15 s to record Fs (steady-state fluorescence signal). Following this, another saturation flash (9,000 μmol m^−2^ s^−1^) was applied and then Fm′ (maximum fluorescence under light-adapted conditions) was determined. Other fluorescent parameters were calculated as follows: the effective PSII quantum yield ΦPSII = (Fm′ − Fs)/Fm′ (Genty et al., 1989); and the non-photochemical quenching coefficient NPQ = (Fm − Fm′)/Fm′. All measurements were carried out in the six plants of each treatment.

### Photosynthesis (CO_2_ fixation)

Leaf photosynthetic rate (Pn; mmol CO_2_/m^2^), transpiration rate, E (mmol/ m^2^s), and stomatal conductance, C (mmol/ m^2^s) were measured in fully expanded leaves (third leaf from apex) with a portable photosynthetic open-system (CI-340, CID, Camas, WA, United States; Schlosser et al., 2012).

Water use efficiency (WUE) was calculated as net photosynthesis (Pn) divided by transpiration (E) as an indicator of stomatal efficiency to maximize photosynthesis, minimizing water loss due to transpiration.

### Photosynthetic pigments: Chlorophylls and carotenoids

Extraction was done according to Porra et al. (1989). One hundred milligram of leaves powdered in liquid nitrogen was dissolved in 1 ml of acetone 80% (v/v), incubated overnight at 4°C, and then centrifuged 5 min at 10,000 rpm in a Hermle Z233 M-2 centrifuge. One milliliter of acetone 80% was added to the supernantant and was mixed with a vortex. Immediately afterwards, absorbance at 647, 663, and 470 nm was measured on a Biomate 5 spectrophotometer to calculate chlorophyll a, chlorophyll b, and carotenoids (xanthophylls and carotenes) using the formulas indicated below (Lichtenthaler, 1987; Porra et al., 1989).


$$\begin{array}{c} Chl\;a\;\left(\mu g/g\;FW\right)=\left[\left(12.25\times Abs\;663\right)-\left(2.55\times Abs\;647\right)\right]\\ \times V\left(mL\right)/weight\;\left(g\right) \end{array}$$

$$\begin{array}{c} Chl\;b\;\left(\mu g/g\;FW\right)=\left[\left(20.31\times Abs\;647\right)-\left(4.91\times Abs\;663\right)\right]\\ \times V\left(mL\right)/weight\;\left(g\right) \end{array}$$

$$\begin{array}{cc} \begin{array}{l} Carotenoids\\ \;\left(\mu g/g\;FW\right) \end{array} & \begin{array}{c} =\left[\begin{array}{l} \left(1000\times Abs\;470\right)-\left(1.82\times Chl\;a\right)\\ -\left(85.02\times Chl\;b\right)/198 \end{array}\right]\\ \times V\left(mL\right)/weight\;\left(g\right) \end{array} \end{array}$$

Tubes were protected from light throughout the whole process.

### Total phenols

Leaf extracts were prepared from 0.25 g of leaves (powdered in liquid nitrogen) in 2.25 ml methanol 80%, sonicated for 10 min, and centrifuged for 5 min at 5,000 rpm.

Total phenols were quantitatively determined with Folin–Ciocalteu agent (Sigma. Aldrich, St Louis, MO, United States) by a colorimetric method described by Xu and Chang (2007), with some modifications. Twenty microliters of extract was mixed with 0.250 ml of Folin–Ciocalteu 2 N and 0.75 ml of Na_2_CO_3_ 20% solution. After 30 min at room temperature, absorbance was measured at 760 nm. Gallic acid was used as standard (Sigma-Aldrich, St Louis, MO, United States); a calibration curve was made (*r* = 0.99). Results are expressed in mg of gallic acid equivalents per 100 g of fresh weight (FW).

### Bioactive quantification by UHPLC–MS

Two hundred and fifty milligram of each powdered sample was weighed into a tube and 2.25 ml of methanol–water (80:20, v/v) were added. Then, the mixture was vortexed and sonicated for 30 min in an ultrasonic bath. The resulting extract was centrifuged for 5 min at 5,974 *g*, the supernatant was collected and the residue was re-extracted again following the same procedure as above. Both supernatants were pooled and evaporated to dryness under reduced pressure at 35°C in a rotavapor R-210 (Buchi Labortechnik AG, Flawil, Switzerland). Next, the residue was reconstituted with 5 ml methanol, filtered through a 0.22 μm NylafloTM nylon membrane filter from Pall Corporation (Ann Arbor, MI, United States), and subsequently analyzed (or stored in a freezer below −20°C prior to analysis). Each sample was prepared in triplicate. Every sample was extracted and analyzed by UHPLC–MS. According to Olmo-García et al. (2018), analyses were done at SIDI.^1^

### RNA extraction and RT-qPCR analysis

Prior to RNA extraction, samples were removed from the −80°C freezer and ground to a fine powder with liquid nitrogen using a sterilized mortar and pestle. Total RNA was isolated from each replicate with GeneJET Plant RNA Purification Mini Kit (Thermo Scientific; DNase treatment included) and after confirmation of RNA integrity using NanodropTM, a retrotranscription followed by a RT-qPCR was performed. This analysis was performed only in controls and H47, as this strain caused the highest increases in bioactives.

The retrotranscription was performed using iScript tm cDNA Synthesis Kit (Bio-Rad). All retrotranscriptions were performed using a GeneAmp PCR System 2700 (Applied Bio-systems): 5 min 25°C, 30 min 42°C, and 5 min 85°C and hold at 4°C. Amplification was performed with a MiniOpticon Real-Time PCR System (Bio-Rad): 3 min at 95°C and then 39 cycles consisting of 15 s at 95°C, 30 s at 55°C, and 30 s at 72°C, followed by melting curve to check the results. To describe the expression obtained in the analysis, cycle threshold (Ct) was used. Standard curves were calculated for each gene, and the efficiency value ranged between 80 and 120%. The reference gene used was GADPH2. Key genes controlling the shikimate-flavonol pathway and DOXP pathway were studied and primers used for each appear in Supplementary Table 1 of Supplementary material. Primers for Flavonol-3-hydroxylase(*OeF3H*), Flavonol-3′-hydroxylase (*OeF3’H*), and Flavonol synthase (*OeFLASYN*) were obtained from Iaria et al. (2016). Primers for Chorismate mutase (*OeCHOMU*; XM_ 023023569.1), Chalcone synthase (*OeCHASIN*; XM_023018868.1), Chalcone isomerase (*OeCHAISO*; XM_023011594.1), Arogenate dehydrogenase (*OeARODESHIDRO*; XM_022989811.1), 1-deoxy-D-xylulose synthase (*OeDOXP*; XM_022992625.1), 8-hydroxigeraniol synthase (*Oe8HYDROXY*; XM_022988413.1), Iridoid synthase (*OeIRISY2*; KX944708.1), and Secologanin synthase (*OeSECSIN*; KX944713.1) were designed on PRIMER3 based on genomes from *Olea europaea*. Var sylvestris and *Olea europaea*. Var koronieki. Results for gene expression were expressed as a differential expression according to Pfaffl (2001). Control expression is set at 1; therefore, only changes above 1 are considered.

### ACE inhibitory activity determination through HPLC

Extract preparation is described in the section “Bioactive quantification by UHPLC-MS.”

The reaction measured was the transformation of the substrate hyppuryl-histidyl-leucine (HHL) into the product hippuric acid (HA), catalyzed by the ACE. The purpose behind this experiment was to determine the inhibitory capacities of the different extracts (same as the one described for the previous analysis) by measuring both the substrate and the product concentrations.

The protocol used was based on the work of Wu et al. (2002) although modifications were performed for its optimisation. Agilent 1100 series equipment was used. Samples were prepared with 20 μl of ACE, 20 μl of HHL (enzyme’s substrate), 40 μl of sample (ACE inhibitor), and 40 μl of borate buffer. Oleuropein at 25 ppm concentration was used as the reference ACE inhibitor in the negative control, and 80% methanol was used instead for the positive control. A column C18 100A 150 × 4.6 mm 5 micron was used, with a 0.5 ml/min flow of the mobile phase (75% miliQ water, 0.1%TFA, and 25% acetonitrile), running for 21 min. A UV sensor was used, set at λ = 226 nm.

The % of inhibition was calculated using the formula:


$$\%inhibition=\frac{A-B}{A}\times \mathrm{100}$$

Where A is the area under the curve (AUC) of the HA peak without ACE inhibitor (C+) and B is HA AUC when ACE inhibitors are added (samples).

### Statistics

To evaluate treatment effects, one way *t*-student (Statgraphics Centurion XVIII) were performed for each of the variables.

## Results

**Figure 1:**
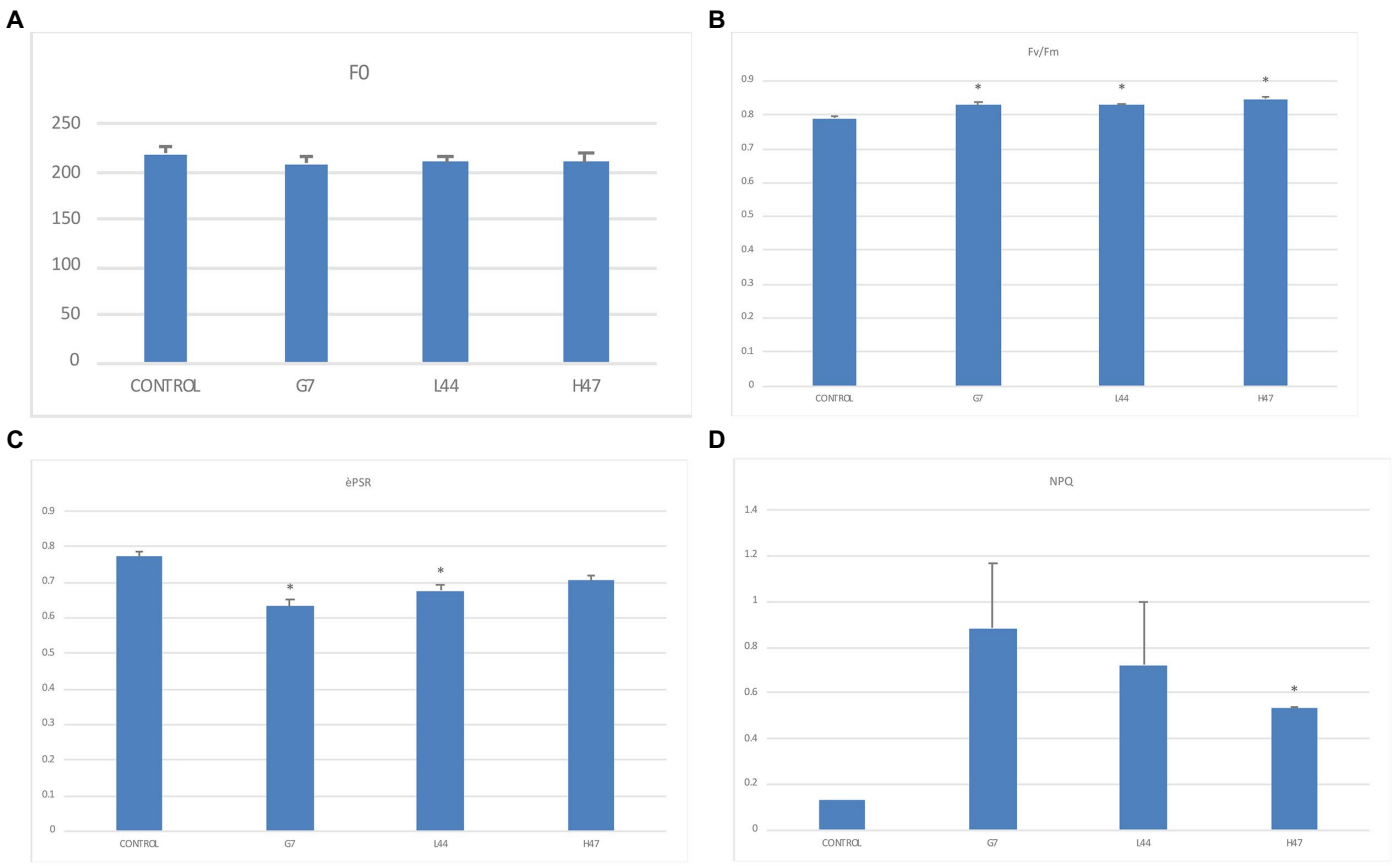
Photosynthetic parameters related to photosystems and light reactions in inoculated and non-inoculated controls. **(A)** Minimal fluorescence after 20-min dark-adaptation (F_o_). **(B)** Maximal PSII quantum yield (Fv/Fm), **(C)** effective PSII quantum yield (ΦPSII), and **(D)** non-photochemical quenching coefficient (NPQ) measured in olive tree plants. For each treatment and parameter average value ±SE value (*n* = 6) is presented. Asterisks (*) represent significant differences between each treatment and the control according to *T* student test (*p* < 0.05).

Photosynthesis (Figure 1) was improved by the three strains. All three decreased F_0_, the minimum fluorescence of adaptation to darkness, reaching similar values under all 3 (Figure 1A), and they also increased the maximum potential photosynthetic capacity of PSII (Fv/Fm) that reached optimal values (0.82–0.85) in inoculated plantlets, while controls were below optimal values (Figure 1B). Efficiency of PSII (ePSR; Figure 1C) was decreased by all three strains although only significantly with G7 and L44, being G7 the lowest; finally, energy dissipation (NPQ) was enhanced by all 3, being significant only by H47 (Figure 1D).

**Figure 2:**
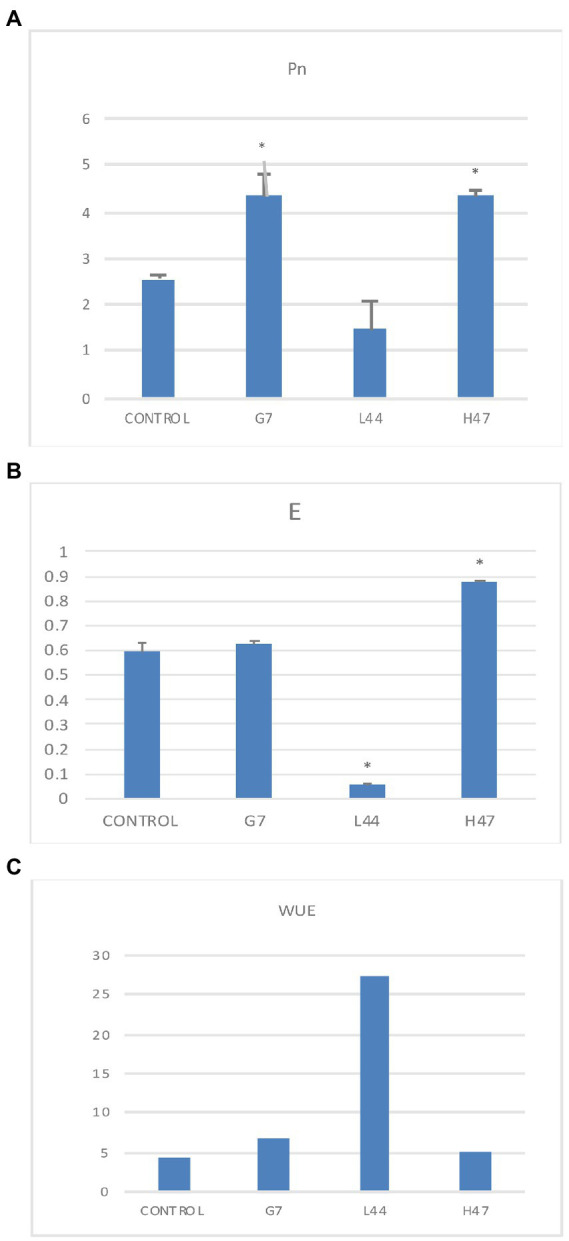
Photosynthetic parameters related to C fixation measured in olive tree plants treated with the three strains and non-inoculated controls. **(A)** Net photosynthesis (Pn) measured as the CO_2_ fixed by the leaves (μmol CO_2_/m^2^s). **(B)** Transpiration rate (E), measured as the amount of water released by transpiration (μmol H_2_O/m^2^s), and **(C)** Water Use Efficiency (WUE) calculated as Pn divided by transpiration rate. Average values of the replicates with SE bars are represented (*n* = 6). Asterisks (*) represent significant differences with the control according to *T* student test (*p* < 0.05).

Net carbon fixation (Figure 2A) in control plants was 2.6 μmol CO_2_ /m^2^ s. This parameter was significantly increased a rough 60% by two strains G7 and H47. As regard to transpiration, control plants averaged 0.6 μmol H_2_O /m^2^ s; only H47 increased transpiration significantly (+46%), while L44 decreased significantly (Figure 2B), dramatically increasing water use efficiency (Figure 2C).

**Figure 3:**
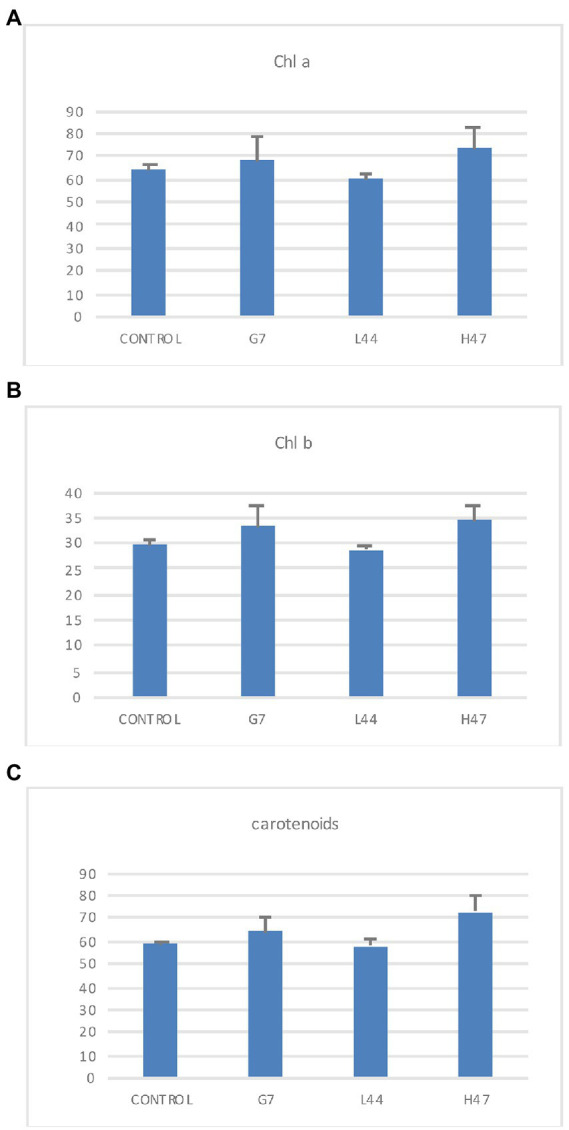
Photosynthetic pigments concentration (μg/g FW). **(A)** Chlorophyll a, **(B)** Chlorophyll b, and **(C)** Carotenoids measured in olive tree leaves treated with the three strains. For each treatment and parameter average value ±SE value is presented (*n* = 6). Asterisks (*) represent significant differences with the control according to the T student test (*p* < 0.05).

Photosynthetic pigments in controls showed similar values of chlorophyll a and carotenoids (65 and 60 μg/g FW, respectively) while chlorophyll b averaged 29 μg/g FW. Bacterial treatments did not cause significant changes, although G7 and H47 slightly increased chlorophylls and carotenes (Figure 3).

**Figure 4:**
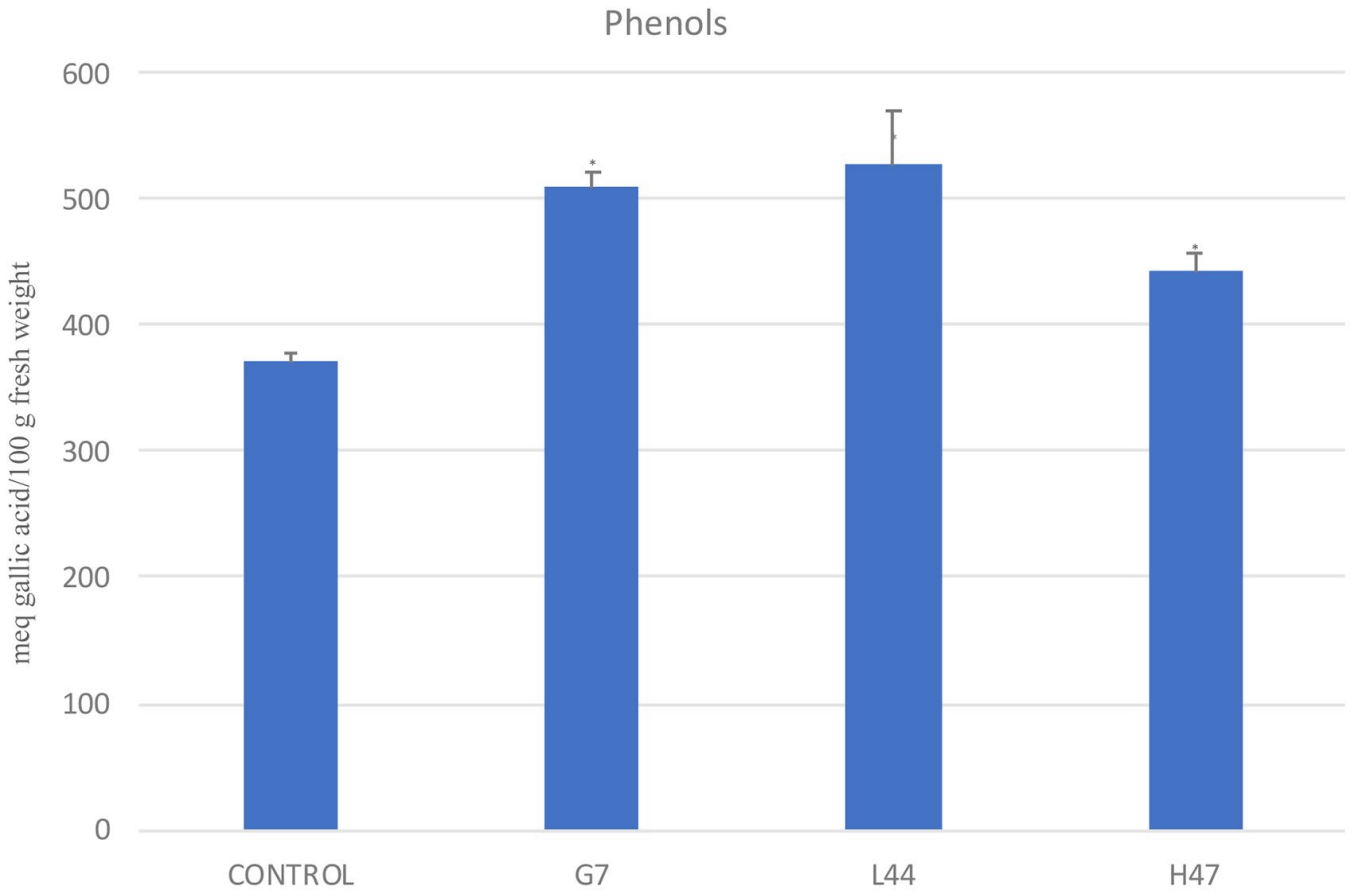
Total phenols (meq gallic acid/100 g fresh weight), in olive tree leaves treated with the three strains and non-inoculated controls. For each treatment and parameter average value ± SE value is presented (*n* = 6). Asterisks (*) represent significant differences with the control according to the T student test (*p* < 0.05).

Total phenols (Figure 4) in controls averaged 370 meq gallic acid/100 g fresh weight. Phenols were significantly enhanced by all three strains, ranging from 19% (H47) to 37% (G7, L44) increases.

**Figure 5:**
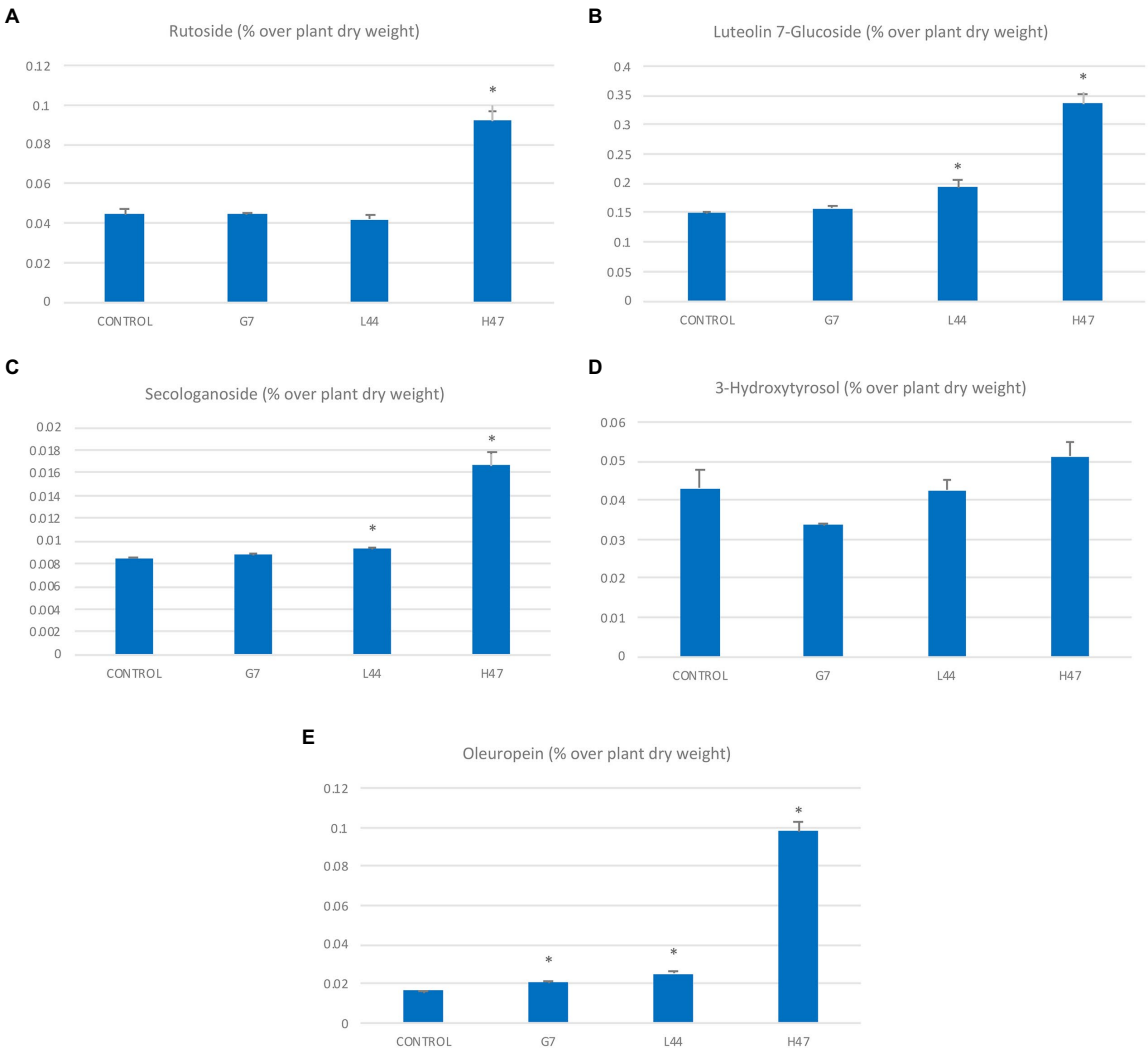
Polyphenol concentration (μg/g DW) in olive leaves from plants treated with the three strains and non-inoculated controls. Flavonols **(A)** Rutoside (μg/g); **(B)** Luteolin 7-glucoside; iridoids **(C)** Secologanoside (μg/g); **(D)** 3-hydroxytirosol (μg/g); and **(E)** Oleuropein (μg/g). For each treatment and parameter average value ± SE value is presented (*n* = 6). Asterisks (*) represent significant differences with the control according to the *T* student test (*p* < 0.05).

The concentration of the most abundant flavonoids, rutoside, and luteolin-7-glucoside in controls was 0.042 and 0.15 μg/g, while secologanoside and oleuropein 0.085 and 0.18 μg/g respectively, and hydroxytirosol was 0.045 μg/g (Figure 5). Flavonoid concentration was increased by 2-fold under the influence of H47, as well as secologanoside (Figure 5C) and oleuropein (Figure 5E) with increases of 2-fold and 8-fold, respectively. Hydroxytyrosol was also enhanced by H47 but not significantly (Figure 5D). G7 and L44 caused mild increases in oleuropein, G7 decreased hydroxytirosol and L44 increased luteolin-7-glucoside.

**Figure 6:**
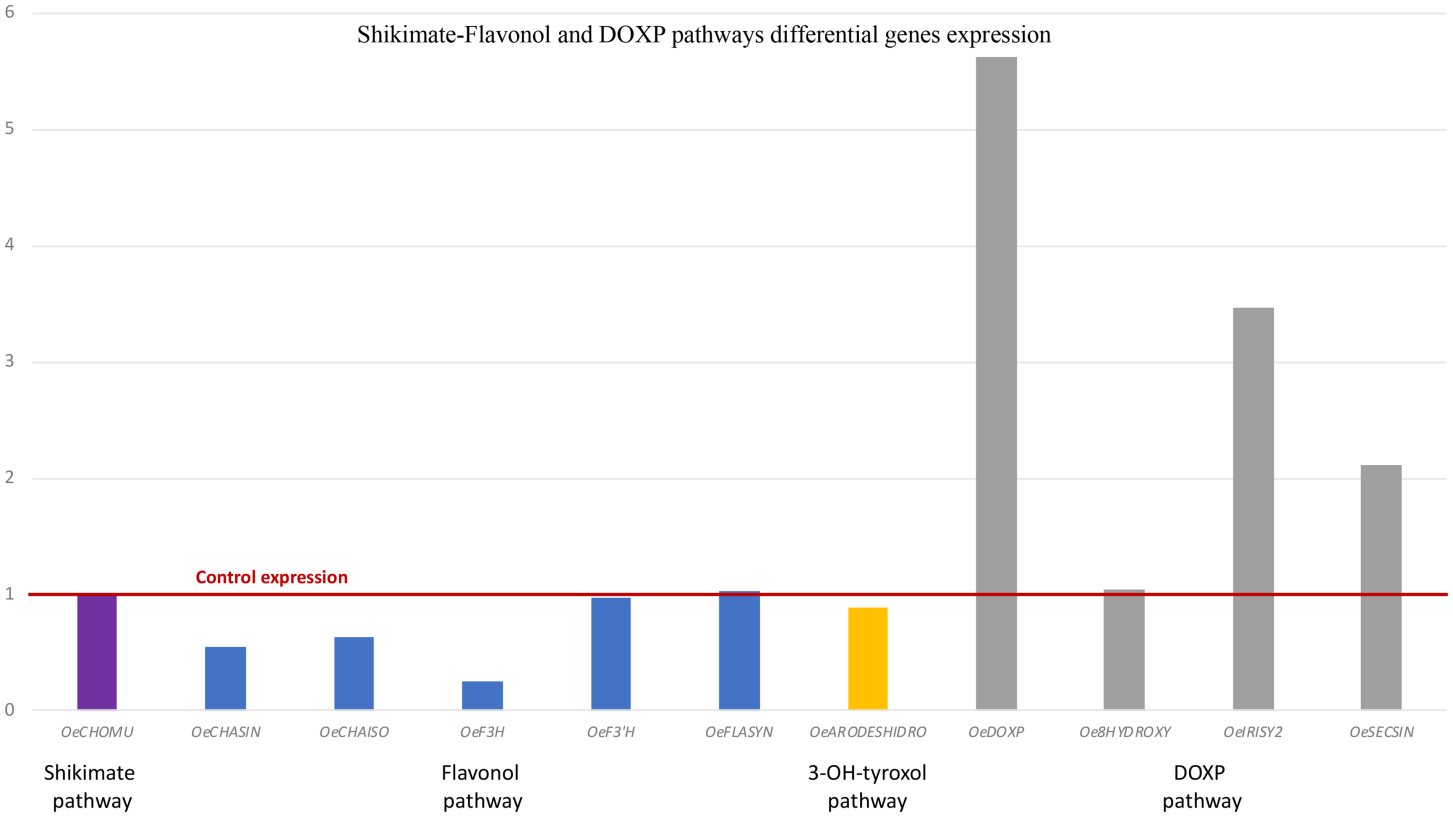
Differential gene expression of genes in the Shikimate-flavonol and DOXP pathways by RT-qPCR. Shikimate pathway (purple bars): Chorismate mutase (*OeCHOMU*); flavonol pathway (blue bars): Chalcone synthase (*OeCHASIN*), Chalcone isomerase (*OeCHAISO*) Flavonol-3-hydroxylase (*OeF3H*), Flavonol-3′-hydroxylase (*OeF3*′*H*), and Flavonol synthase (*OeFLS*); 3-OH-tyroxol pathway (yellow bars): Arogenate deshidrogenase (*OeARODESHIDRO*); DOXP pathway (gray bars): 1-deoxy-D-xylulose synthase (*OeDOXP*), 8-hydroxygeraniol synthase (*Oe8HYDROXY*), Iridoid synthase (*OeIRYSY2*), and Secologanin synthase (*OeSECSIN*).

The expression of key genes controlling the shikimate-flavonol pathway and DOXP pathway was analyzed by RT-qPCR in controls and in H47-treated plants, as this strain showed the highest increases in bioactive metabolites (Figure 6). Shikimate pathway control point, Chorismate mutase, was similarly expressed in both treatments. In the flavonol-pathway, Chalcone synthase, Chalcone isomerase, and Flavonol-3-hydroxylase were downregulated, and F-3’hydroxylase and Flavonol synthase were evenly expressed; arogenate dehydrogenase was slightly downregulated. As regards to enzymes in the DOXP pathway, all three synthases were upregulated, being DOXP synthase the most affected (8-fold), iridoid synthase by 3.5-fold and secoiridoid-synthase by 2-fold.

**Figure 7:**
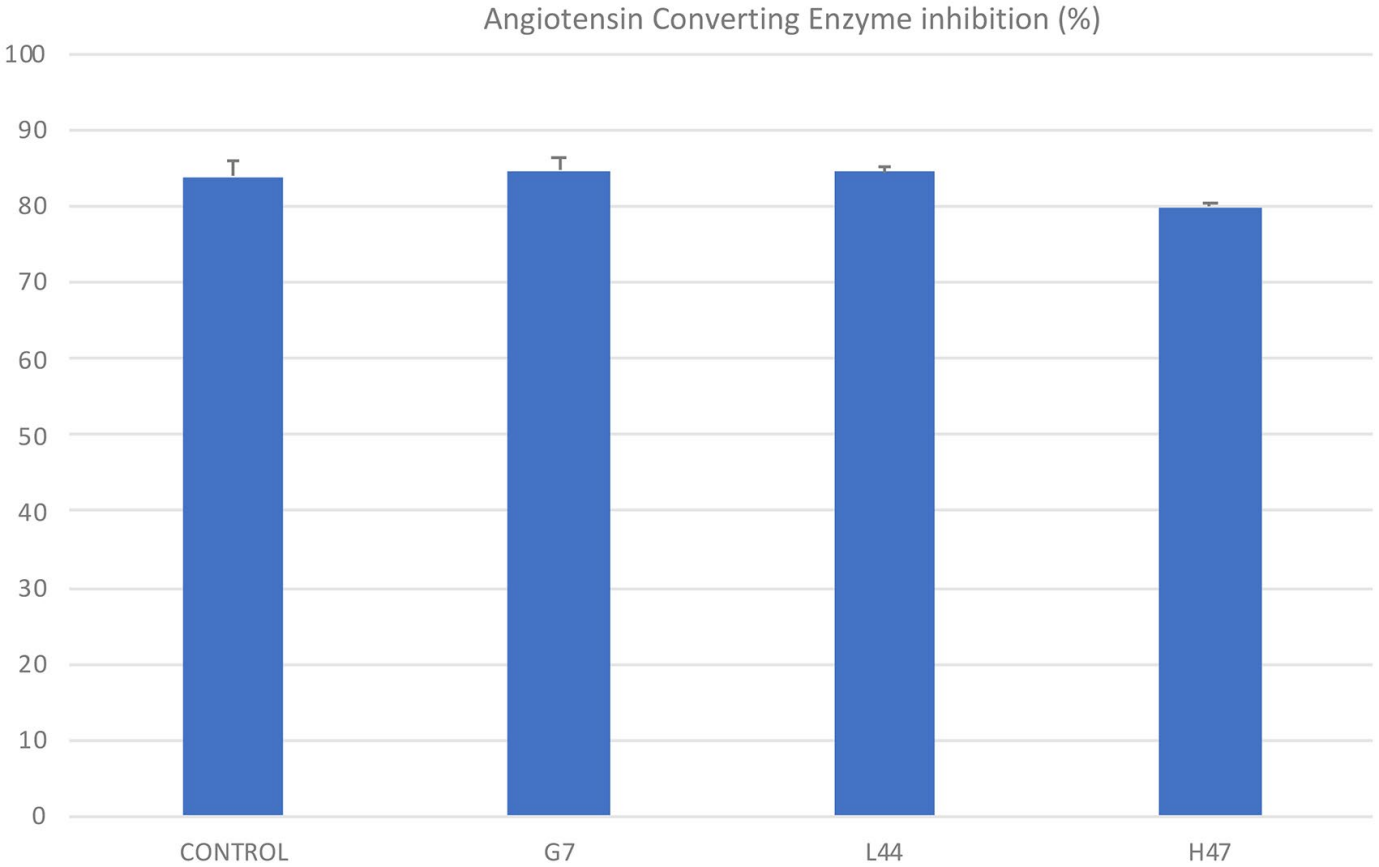
Angiotensin Converting Enzyme inhibition (%) by olive leaf extracts obtained from inoculated (G7, L44, and H47) and non-inoculated controls. For each treatment and parameter average value ± SE value is presented (*n* = 6). Asterisks indicate significant differences, according to *T* student test (*p* < 0.05).

The ability of extracts from the three strains and the non-inoculated controls to inhibit Angiotensin Converting enzyme was evaluated (Figure 7). Only H47 slightly decreased olive leaf extracts inhibitory potential.

## Discussion

The ability of certain PGPBs to trigger plant metabolism has been widely reported and still, specificity of plant-bacterium and specific targets triggered by each bacterial strain continue to be a challenge of interest. All three strains were able to trigger olive metabolism differently reinforcing the high specificity of the plant-microbe interactions on one hand, and the multitarget potential of each strain to improve adaptation. Furthermore, potential of PGPB to improve plant adaptation was potentiated by the harsh growth conditions assayed, sampling after summer period when extremely high temperatures called for bacterial induced benefits.

Photosynthesis determined by fluorescence indicated that the three *Bacillus* strains were able to improve plant fitness, bringing (Fv/Fm) to optimal values and decreasing basal stress according to F_o_ values (Lucena et al., 2012). The decrease of PSII efficiency (ePSR; Figure 1C) together with the significant increase of energy dissipation support the ability of the three strains to alleviate oxidative stress induced by excess UV radiation and water stress, enabling a better use of resources (Ilangumaran and Smith, 2017; Sachdev et al., 2021; Gupta et al., 2022).

Net carbon fixation (Figure 2) was differently modified by each *Bacillus* strain. L44 dramatically decreased transpiration suggesting a protective mechanism based on keeping water by closing stomata to prevent its loss, increasing WUE, which limits CO_2_ fixation (Jones and Sutherland, 1991; Aires et al., 2022). G7 increased CO_2_ fixation not altering transpiration, suggesting that metabolic changes that allow improved water potential happened. Only H47 increased transpiration significantly, improving plant adaptation by allowing the plant to keep stomata opened while keeping an active C fixation, hence improving C fixation (Hura et al., 2007); the improvement in CO_2_ assimilation together with the improvement in light energy absorption support the increase in secondary metabolites in this case, instead of the reported tomato yield increase (Aires et al., 2022).

**Figure 8:**
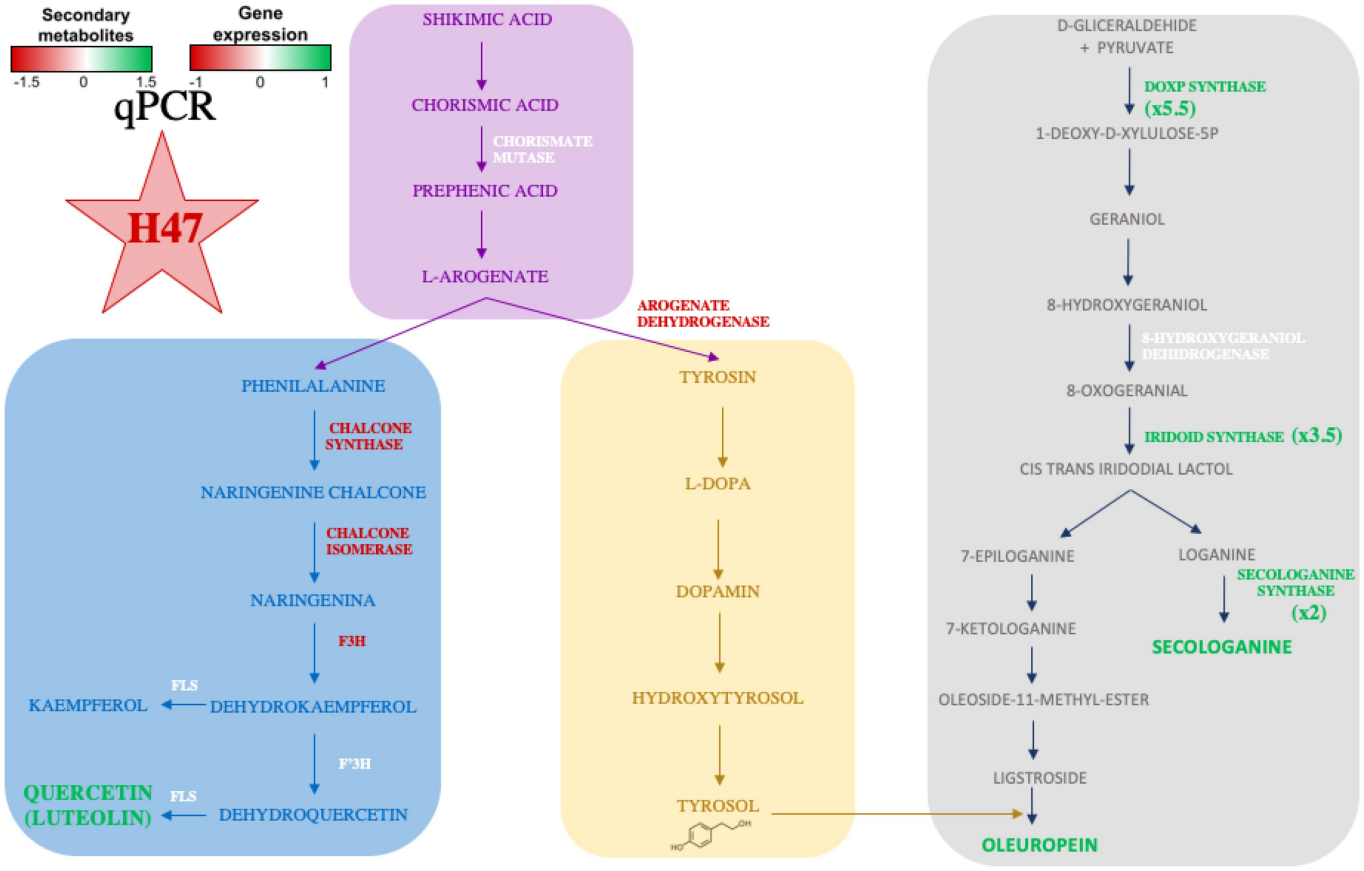
Representation of the shikimate-flavonol pathway, the phe and tyr branches leading to main flavonols or tyrosol, respectively and DOXP-pathway with studied enzymes. Names of enzymes (and metabolites) in green indicate higher concentration/expression, in red are lower and in white do not change.

Effects on photosynthetic parameters reveal the different targets that each strain is able to trigger in order to improve plant adaptation (Ilangumaran and Smith, 2017). Interestingly, Fv/Fm is a common target to all strains, despite the different mechanism used by each of them; it confirms enhanced plant fitness under water and salt stress as it reaches standard values for healthy plants. Also, total phenols are increased by all three strains, revealing activation of antioxidant molecules to cope with oxidative stress due to environmental conditions (Alsayed et al., 2012). Enhancement of secondary metabolites such as polyphenols upon PGPB delivery, bacterial or chemical elicitors such as salicylic acid have been widely reported (Figure 8; Preciado-Rangel et al., 2019; Gutierrez-Albanchez et al., 2020; Martin-Rivilla et al., 2021).

Despite the increase in total phenols, HPLC-MS analysis revealed different profiles on major secondary metabolites present in olive leaves induced by PGPBs, being H47 outstanding in terms of concentration. The striking 2-fold increases in flavonols and in secologanoside, and the 5-fold increase in oleuropein (Figure 5) were further studied by exploring changes in gene expression (Figure 6) in the shikimate-flavonol pathway leading to flavonols and to hydroxytirosol, and the DOXP pathway, leading to the iridoids secologanoside, ligstroside, and oleuropein (Figure 8). The biosynthetic pathway to iridoids in olive has been unveiled in the last years to describe oleuropein synthesis to follow an independent pathway from secologanoside. The early steps of iridoid synthesis result in 8-oxogeranial, substrate to iridoid synthase, that is transformed into 7-ketologanin in several steps and finally in oleoside 11-methyl ester by OeOMES, which will finally be transformed into ligstroside to be condensed with 3-hydroxytirosol to release OLE (Koudounas et al., 2021); Rodríguez-López et al. (2021) demonstrated that is independent of secoxyloganin in *Olea europaea*.

The shikimate pathway has been widely studied as it is central to plant’s metabolism, leading to aromatic aminoacids trp, phe, and tyr (Oliva et al., 2021). After addition of side chain by enol-pyruvyl-shikimate synthase, the first control point is on Chorismate mutase and determines trp or phe/tyr synthesis; accumulation of either product inhibits the enzyme. Next control point is on L-arogenate processing enzymes, as arogenate dehydratase will lead to phe, head of flavonoids, while arogenate dehydrogenase will determine tyrosin biosynthesis, leading to 3-hydroxy-tyrosol, one moiety of oleuropein (Supplementary Table 2 of Supplementary material). In this metabolic pathway, accumulation of end-products inhibits branching point enzymes (Maeda and Dudareva, 2012; Oliva et al., 2021). *Bacillus* H47 kept the shikimate pathway active, as Chorismate mutase was similarly expressed in both groups of plants (Figure 6) despite differential accumulation of target metabolites (Figure 5) which did not play an inhibitory role, suggesting additional control of the pathway by H47, probably blocking expression of transcription factors in charge of this inhibition, such as MYB4 (Garcia-Seco et al., 2015; Gutierrez-Albanchez et al., 2020).

Despite the higher accumulation of flavonoids in H47 treated plants (Figure 5), expression of enzymes involved in flavonol synthesis was not enhanced as compared to controls (Figure 6). This fact has been described before (Gutierrez-Albanchez et al., 2020) and is consistent with dynamics of plant innate immunity, for which the “network pulse model” has been proposed (Kollist et al., 2019). In this model, the response is quickly triggered upon stress to activate systemically plant’s immune system and integrated into the whole plant by pulses of gene expression, registering peaks and valleys on expression, with responses that run from seconds to minutes, to hours (Kollist et al., 2019). Interestingly, this pulse-powered response has been also described to control rapid stomatal responses, enabling a fine-tunning response of adaptation to water stress (Melotto et al., 2006) keeping high rates of C assimilation; the simultaneous enhancement of flavonol concentration with lower expression and higher transpiration (Figure 2), supports the regulation of the response through the pulse model network (Kollist et al., 2019). Furthermore, H47 seems to be controlling carbon allocation preferentially to secondary metabolites under extreme adverse conditions, further exacerbated by summer temperatures (Mauch-Mani et al., 2017). According to this hypothesis, G7 and L44 could have been sampled on valleys of signal transduction, but effects are not shown on the secondary metabolites evaluated, so bacterial activation targets fall out of focus set in this case.

On the other hand, the DOXP pathway was also activated, and there is a consistent accumulation of secologanoside (Figure 5) with expression of the key synthases in the pathway.

Interestingly, an 8-fold increase of the first enzyme in this pathway DOXP-synthase is detected, while intermediate synthases exclusive to iridoid synthesis, are only 3.5-and 2-fold. The enhanced activation of DOXP-synthase speaks of an activation devoted to feed synthesis of other relevant terpenes with physiological or biological activity, such as ABA, key for stomatal control, or oleacin synthesis (El and Karakaya, 2009; Li et al., 2020; Skodra et al., 2021; Xie et al., 2021). A striking 5-fold increase in the target metabolite oleuropein was found, suggesting either activation of the final step in the pathway, or a strong inhibition of oleuropein-β-glucosidase (OeGLU), the enzyme that would transform oleuropein into its aglycon, which would be further transformed into oleacin by demethylation and decarboxylation (Koudounas et al., 2021). However, as transitory silencing of *OeGLU* results in secoiridoid biosynthesis arrest (Koudounas et al., 2021), which is not the case in this study, the activation of the final step by H47 seems more likely to explain oleuropein increases. The increased expression of OeIRISY2 (Figure 6) may be masking expression of oleoside-methyl ester synthase (OMES), as OeSECSIN cannot differentiate the amplification of OMES2 (genbank nr MT909124.1) and OMES (oleoside methyl ester synthase; genbank nr MT909123.1). They hybridize 100% to both genes. Also they do not hybridize to secoxyloganin synthase (SXS genbank nr. MT909125).

Oleuropein is an interesting polyphenol accumulated in leaves, where it plays a role as antioxidant and osmolyte, contributing to plant adaptation to water stress (Mechri et al., 2020) and involved in plant defense to pathogens (Benyelles et al., 2014) and to hervibores (Koudounas et al., 2021). Cytological accumulation occurs in vacuoles so activation of specific oleuropein carriers to vacuoles must be involved in the increase (Ghimire et al., 2021). Therefore, the reported increase in leaves by H47 contributes to improved plant adaptation modifying water potential, allowing more efficient water absorption, and probably contributing to the enhanced transpiration detected.

Interestingly, oleuropein is also a beneficial molecule for humans, as it is one of the bioactive molecules contributing with antihypertensive effect (Romani et al., 2019), so the potential of leaf extracts enriched in oleuropein were expected to show increased antihypertensive effect. To test our hypothesis, the ability to inhibit the Angiotensin Converting enzyme as an *in vitro* marker of this activity was evaluated. Opposite to our expectations, the inhibition of ACE activity was even lower than controls (Figure 7), which could be explained on one hand, because the molecule directly inhibiting ACE is oleacin, another iridoid, which concentration may be lower due to preferential accumulation of oleuropein, or, on the other hand, because oleacin might be translocated to fruits, where it would preferentially accumulate, accounting for the benefits of olive oil, as previously reported for flavonols in blackberry and tomato (Gutierrez-Albanchez et al., 2021; Aires et al., 2022).

## Conclusion

In conclusion, *Bacillus* H47 improved plant photosynthetic efficiency and C assimilation by keeping stomata open; simultaneous activation of secondary metabolites biosynthesis at DOXP and shikimate pathways resulted in increased flavonol and iridoid concentration in leaves, although the antihypertensive activity was not enhanced.

## Data availability statement

The original contributions presented in the study are included in the article/**Supplementary material**, further inquiries can be directed to the corresponding author. Data is available upon request.

## Author contributions

FG-M, AG-V, and BR-S: conceptualization. EG-C and MM-P: formal analysis. FG-M: resources. AG-V, MM-P, and BR-S: data curation. EG-C: writing—original draft preparation. BR-S and AG-V: writing—review and editing. FG-M: supervision. FG-M and BR-S: funding acquisition. All authors contributed to the article and approved the submitted version.

## Conflict of interest

The authors declare that the research was conducted in the absence of any commercial or financial relationships that could be construed as a potential conflict of interest.

## Publisher’s note

All claims expressed in this article are solely those of the authors and do not necessarily represent those of their affiliated organizations, or those of the publisher, the editors and the reviewers. Any product that may be evaluated in this article, or claim that may be made by its manufacturer, is not guaranteed or endorsed by the publisher.

## Footnotes

^1^ https://www.uam.es/uam/sidi
