# Supplementary material for: Bacillus H47 triggers Olea europaea metabolism activating DOXP and shikimate pathways simultaneously and modifying leaf extracts’ antihypertensive activity
Source: Front Microbiol. 2022 Oct 4;13:1005865. doi: 10.3389/fmicb.2022.1005865 (PMC9577608; doi:10.3389/fmicb.2022.1005865)
Supplement: Supplementary file 1 [file Data_Sheet_1.docx]

Supplementary Material

**Table S.1.** Primers used for RT-qPCR expression analysis

| **Gene Identifier** | **Gene name** | **Forward Primer** | **Reverse Primer** |
| --- | --- | --- | --- |
| *OeCHOMU* | *Chorismate mutase* (XM_023023569.1) | 5’GCCGCAACTTGTGACACAAT | 5’GGGAGGCTCGGTATTTTGCT |
| *OeCHASIN* | *Chalcone synthase* (XM_023018868.1) | 5’GAGCGCCTGTGTGTTGTTTA | 5’AAACCCTTCACCACTTGTGC |
| *OeCHAISO* | *Chalcone isomerase* (XM_023011594.1) | 5’CAGGCCAGGTGAGAAGAGAG | 5’CCCAAGTGAAAAGGGGACTT |
| *OeF3H* | *Flavonol-3-hydroxylase*(Iaria et al, 2016) | 5’TCCTCTGCCCGTGTGATAGT | 5’AATCCGTGTGATGCAGTGAG |
| *OeF3'H* | *Flavonol3′hydroxylase*(Iaria et al, 2016) | 5’GTGGCAGAAGCTGACCTACC | 5’CGTAGAGCCCTTTGGAATGA |
| *OeFLASYN* | *Flavonol synthase*(Iaria et al, 2016) | 5’GAATAGAGTCACTGTCAAGGG | 5’TCCCTAACCATCGTGGCCTTT |
| *OeARODESHIDRO* | *Arogenate deshidrogenase* (XM_022989811.1) | 5’ATTCCAGTCTCCCCCTCCTC | 5’TGTGCAAAAGGGCCAAAACC |
| *OeDOXP* | *1-deoxy-D-xylulose synthase* (XM_022992625.1) | 5’CGACAAGCATCTCAGCTGGA | 5’GCAGTCATAGCTCCGTCTCC |
| *Oe8HYDROXY* | *8-hydroxigeraniol synthase* (XM_022988413.1) | 5’TGTGACAAAAGGCCAAATCA | 5’CAGGGCAAATGCAGAACTTT |
| *OeIRISY2* | *Iridoid synthase* (KX944708.1) | 5’TAGCTCGACTGAAGCAACCG | 5’GGTGGACCGACCATGTTAA |
| *OeSECSIN* | *Secologanin synthase* (KX944713.1) | 5’ TGCTGCGCCCTTCATCTTTA | 5’ ACACCTTGGGTCTCAGTCCT |

| **Name** | **Structure** |
| --- | --- |
| Secologanin | 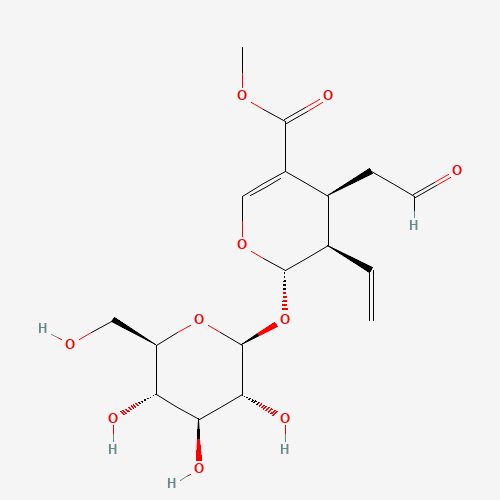 |
| Tyrosol | 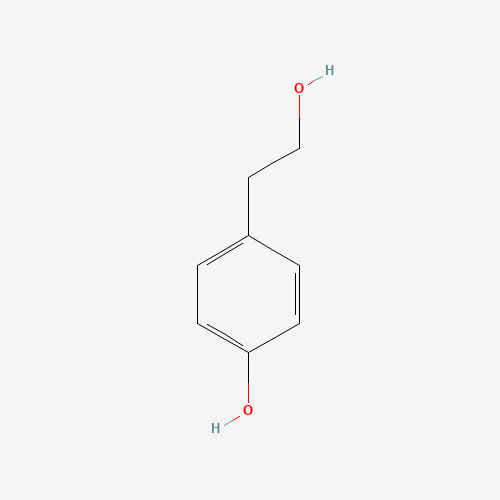 |
| Oleuropein | 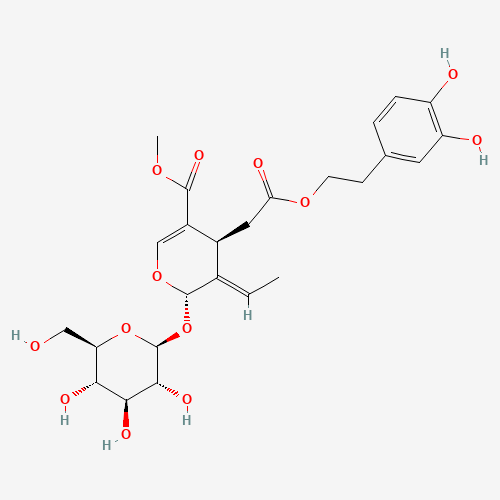 |

**Table S.2.** Molecular structures of Oleuropein, Tyrosol and Secologanin
